# Evaluation of a Methodology for Estimating HbA1c Value by a New Glucose Meter

**DOI:** 10.1177/1932296815587323

**Published:** 2015-05-22

**Authors:** Jochen Sieber, Frank Flacke, Bonnie Dumais, Casey C. Peters, Erin B. Mallery, Liz Taylor

**Affiliations:** 1 Sanofi-Aventis Deutschland GmbH, Global Diabetes Division, Frankfurt am Main, Germany; 2 AgaMatrix, Inc, Salem, NH, USA; 3 MassResearch LLC, Waltham, MA, USA

**Keywords:** eA1c, eA1c questionnaire, estimated HbA1c, MyStar Extra blood glucose meter, self-monitored blood glucose

## Abstract

**Background:**

Accuracy/robustness of HbA1c estimation (eA1c) with an algorithm built into the MyStar Extra blood glucose (BG) meter has been demonstrated by in silico testing. We evaluated the performance and use of eA1c in a clinical setting.

**Methods:**

Subjects took the BG meter home for 4 months to obtain eA1c in this open-label, single-center study. Laboratory HbA1c values were obtained approximately every 2 weeks and the corresponding eA1c documented. Subjects completed a questionnaire at study end (NCT01885546).

**Results:**

There were 133 enrolled subjects (mean [SD] age 60.0 [15.0] years, 69 males, 104 with diabetes, HbA1c 7.0% [1.4]). A total of 1008 pairs of eA1c and laboratory HbA1c values were available. In subjects with diabetes, 97.5% of the eA1c results fell within ±20% of the laboratory HbA1c, 95.0% within ±18%, and 90.7% within ±15%. When results were limited to the reportable HbA1c range of ≥6 to ≤10%, 99.3% of eA1c values fell within ±20% of the laboratory HbA1c, 98.5% within ±18%, and 96.2% within ±15% Most subjects agreed/strongly agreed that the eA1c section in the user guide and flash cards was easy to follow (72%), they would use the system to track their eA1c (70%), they found the eA1c tool helpful (79%), and the tool may motivate them to manage their diabetes better (83%).

**Conclusions:**

Accuracy of the eA1c feature in this clinical setting was similar to the performance in silico. The majority of subjects found this tool helpful and agreed it may motivate to manage their diabetes better.

The use of hemoglobin A1c (HbA1c) for monitoring glucose control in people with diabetes was first proposed in 1976.^1^ HbA1c is now the established standard clinical measurement used as a surrogate marker for average glycemic control.^2^ Self-monitored blood glucose (SMBG) and continuous glucose monitoring data have been used to identify a linear correlation between HbA1c and average glucose values as a means for calculating estimated average glucose for HbA1c values.^3^ However, factors such as SMBG timing and frequency influence how SMBG reflects true underlying average glycemia. To reduce estimation bias, weighted average and nonlinear approaches have been explored.^4^ These methods, while sound in theory, remain impractical, leaving the need for an approach that can provide accurate real-time estimates of HbA1c from infrequent SMBG data.

Kovatchev et al published a method for tracking changes in average glycemia in diabetes, based on a conceptually new approach to the retrieval of SMBG data.^5^ The principal premise of this approach is the understanding of HbA1c fluctuation as the measurable effect of an underlying dynamical system’s action. SMBG provides occasional glimpses at the state of this system and, using these measurements, the hidden underlying system trajectory can be reconstructed for each individual. Using compartmental modeling, they have constructed a new 2-step algorithm that includes: real-time estimates of HbA1c (eA1c) from fasting glucose readings, updated with any new incoming fasting SMBG data point, and initialization and calibration of the estimated HbA1c trace with a daily SMBG 7-point profile taken approximately every month. The model was developed from a training dataset of clinical trial data that contained 17,863 fasting SMBG readings and approximately monthly 7-point profiles as well as corresponding HbA1c measurements for 379 individuals. After using the training data, all formulas were fixed and then applied without modification to a test dataset from the same clinical trial that contained 17,925 fasting SMBG readings and approximately monthly 7-point profiles and corresponding HbA1c measurements for an independent group of another 375 individuals. HbA1c was measured by standard laboratory procedures. The model, which was validated with 95% of the eA1c results falling within 17% of the corresponding laboratory HbA1c values,^5^ is the algorithm within the MyStar Extra® blood glucose (BG) meter (AgaMatrix, Inc, Salem, NH, USA; Sanofi, Paris, France) used to estimate HbA1c. In this report, we evaluated both the performance of eA1c in a clinical setting and also how easy it was to use.

## Methods

### Study Design and Participants

The study protocol was approved by the Western Institutional Review Board (Puyallup, WA, USA), and the study was conducted in accordance with the ethical principles of the Declaration of Helsinki (ClinicalTrials.gov NCT01885546). This was a single-arm, single-blinded study; eligible subjects were ≥18 years old, and either subjects without diabetes or subjects with type 1 or type 2 diabetes mellitus (T2DM) who were being treated with any type of approved medication/therapy. They must have been willing to perform a 7-point glucose profile over the course of 1 day, once a month, to perform fasting BG tests every day, complete a follow-up questionnaire, and consent to baseline and follow-up HbA1c assays. Subjects were excluded if they were pregnant, had a hematocrit outside of the measurement range (<20 or >60), or had any condition that the principal investigator deemed may interfere with the subject’s ability to participate. Written informed consent was obtained from each subject during the screening visit.

Each enrolled subject took a MyStar Extra BG monitoring system kit home for 4 months. They used the owner’s guide from the kit and flashcards that were handed out to obtain eA1c values. The BG meter was designed to record eA1c values of ≥6 to ≤10; values below 6 and greater than 10 were reported as “low” or “high,” respectively. Subjects visited the clinical site approximately every 2 weeks to have their laboratory-drawn HbA1c measured and for the clinical staff to obtain the eA1c value calculated from the subject’s meter. HbA1c was measured using a COBAS Integra 800 instrument (Roche Diagnostics, Mannheim, Germany). Subjects were blinded to the laboratory-drawn HbA1c result as well as the eA1c value calculated by their BG meter to prevent introducing bias—if subjects had known their eA1c value, they may have changed their lifestyle to influence the results. Subjects completed a questionnaire at the end of the study where they were asked whether they strongly agreed, agreed, were neutral, disagreed, or strongly disagreed with 7 statements concerning the concept of eA1c using the MyStar Extra BG meter.

The primary objective of the study was to evaluate the accuracy of the algorithm that was modeled to estimate A1c using the MyStar Extra BG meter compared with laboratory HbA1c. The secondary objective was to evaluate the accuracy of the eA1c trend arrow by comparing the trend of eA1c computed by the BG meter with the trend of laboratory HbA1c.

### Statistical Analysis

To assess the accuracy of eA1c compared with the laboratory HbA1c, the following hypothesis test was performed: H_0_: Pr{accurate result} ≤95% versus the alternative, H_1_: Pr{accurate result} >95%, where an “accurate result” was defined as an individual eA1c falling within ±20% of the laboratory HbA1c. This accuracy estimate was based on the error grid analysis in silico where 97.9% of eA1c values fell with ±20% of the laboratory reference HbA1c.^5^

To assess the accuracy of eA1c trend arrows displayed by the device, the following hypothesis test was performed: H_0_: Pr{trend arrow does not trend opposite to reference} ≤97% versus the alternative, H1: Pr{trend arrow does not trend opposite to reference} >97%, where the reference trend was defined as “up” if laboratory HbA1c increased between measurements by more than 0.3% A1c units per month, “down” if laboratory HbA1c decreased between measurements by more than 0.3% A1c units per month, or were otherwise “level.”

Hypothesis tests were performed on data sets for all subjects as well as only subjects with diabetes, the population intended to use the device. Both tests were *t* tests for proportions, using a significance level of 5%. Results of the questionnaire were tabulated for all subjects.

Due to the dependent nature of the within-subject estimated A1c–laboratory HbA1c pairs, the sample size needed to be adjusted based on the within-subject correlation. The within-subject correlation coefficient was .81 for the 798 estimated A1c–laboratory HbA1c pairs for subjects with diabetes and .88 for the 1008 pairs for all subjects. Each subject contributed on average 8 pairs. Based on these values the effective sample size was determined to be 119 for subjects with diabetes and 141 for all subjects.

## Results

### Subject Disposition and Demographics

Of the 133 eligible subjects, 131 completed the study; 2 subjects withdrew their consent. Demographic characteristics of the subjects are shown in Table 1. The majority of subjects had diabetes (78%), with most having T2DM (60%). The average number of years performing SMBG was 14.8 and the average frequency of SMBG was 3.4 times per day.

**Table 1.** Subject Demographics (*N* = 133).

Age, years, mean (SD)	60.0 (15.0)
Male, n (%)	69 (52)
Nondiabetic subjects, n (%)	29 (22)
Diabetic subjects, n (%)	104 (78)
Type 1	24 (18)
Type 2	80 (60)
SMBG experience, years	14.8 (8.7)
Daily frequency of SMBG, number of times	3.4 (2.3)
HbA1c, %, mean (SD)	7.0 (1.4)
Min; max	5.2; 11.9

SD, standard deviation; SMBG, self-monitored blood glucose.

### eA1c Accuracy

**Figure 1.**
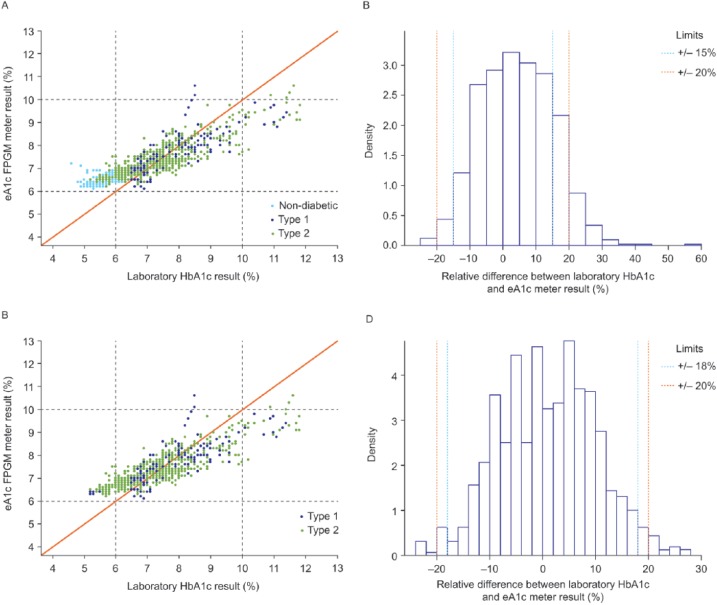
Scatter plot of eA1c versus laboratory HbA1c for all subjects (A) and only subjects with diabetes (C) and histogram of the relative difference between laboratory HbA1c and eA1c for all subjects (B) and only subjects with diabetes (D). The data were stratified by laboratory HbA1c (vertical dashed lines) or eA1c (horizontal dashed lines) values below 6% and above 10%. The eA1c values > 10 are shown because internally the meter continued to calculate the eA1c values regardless of whether or not the value is <6 or >10. Therefore, for accuracy evaluation purposes the actual values are shown. In the field, the meter would show “low” or “high” to the users with values < 6 or >10, respectively.

There were 1064 possible data points, of which 1008 pairs of eA1c and laboratory HbA1c values were available for the analysis. This data set was representative of all 133 subjects adjusted for missed data due to errors and withdrawal of consent. Figure 1A shows the scatter plot of eA1c versus laboratory HbA1c for all subjects. Mean (95% confidence interval [CI]) slope was 0.52 (0.50-0.53) and mean (95% CI) intercept was 0.04 (0.03-0.04). A total of 92.6% of eA1c values fell within ±20% of the laboratory HbA1c, 87.2% within ±18%, and 79.6% within ±15% (Figure 1B). There were 728 (72.2%) of the 1008 pairs in the reportable HbA1c range of 6 to 10% of the BG meter (range of estimated values displayed by the meter). When the results were limited to this range, 99.3% of eA1c values fell within ±20% of the laboratory HbA1c, 98.5% within ±18%, and 96.2% within ±15% (Figure 1A).

Figure 1C shows the scatter plot for the 798 pairs of data from subjects with diabetes. Mean (95% CI) slope was 0.53 (0.51-0.55) and mean (95% CI) intercept was 0.03 (0.03-0.04). A total of 97.5% of eA1c values fell within ±20% of the laboratory HbA1c, 95.0% within ±18%, and 90.7% within ±15% (Figure 1D). A total of 711 (89%) of the 798 data pairs from subjects with diabetes were in the reportable HbA1c range of 6 to 10%. When the results were limited to this range, 99.3% of eA1c values fell within ±20% of the laboratory HbA1c, 98.5% within ±18%, and 96.2% within ±15% (Figure 1C).

Greater than 95% of eA1c values were within ±20% of the laboratory HbA1c for subjects with diabetes, as the 95% CI lower limit was 95.1%. For all subjects, the 95% CI lower limit was 92.6%.

### Accuracy of eA1c Trend Arrows

The hypothesis that at least 97% of trend arrows displayed were steady or in the same direction as the change in reference HbA1c in the reportable range of ≥6 to ≤10 was tested. The analysis indicated that the specified acceptance criterion was not met. However, 95.5% of the trend arrows for all subjects and 95.4% for only subjects with diabetes did not trend opposite to the reference.

### eA1c Questionnaire

**Figure 2.**
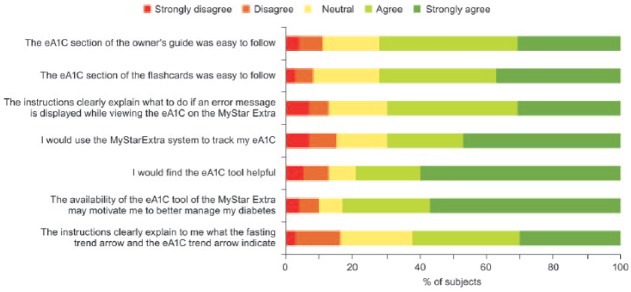
eA1c questionnaire results.

A questionnaire was used to evaluate the subject’s understanding of the concept of eA1c as well as the trend arrows (Figure 2). A total of 72% of subjects agreed or strongly agreed that the eA1c section in the user guide as well as the flash cards were easy to follow. In all, 70% agreed or strongly agreed that they would use the system to track their eA1c and 79% agreed or strongly agreed that they find the eA1c tool helpful and 83% agreed or strongly agreed that the tool may motivate them to manage their diabetes better. A total of 60% agreed or strongly agreed that the instructions clearly explain what the fasting BG trend arrow and the eA1c arrow indicate.

## Discussion

The goal for people with diabetes is to maintain adequate glycemic control to reduce the risk of microvascular and macrovascular complications. Daily monitoring of BG levels provides a point estimate of glycemic control, but HbA1c is recognized as the gold standard marker for average glycemia over time. However, HbA1c assays are typically performed in a laboratory only every few months. Thus, a method is needed to track average glycemia during the time between laboratory measures. SMBG offers such a possibility. Several approaches have been used to correlate SMBG and/or continuous glucose monitoring data and HbA1c,^3,4^ but none have proved to be practical. Recently, Kovatchev et al have constructed a new 2-step algorithm that includes midterm eA1c from fasting glucose readings, updated with any new incoming fasting SMBG data point, and initialization and calibration of the estimated A1c trace with daily SMBG profiles taken approximately every month.^5^ The algorithm was validated in silico, using data from a trial with patients with T2DM, and 95% of the results fell within 17% of the corresponding laboratory HbA1c value. It is intended as an adjunctive tool to complement, not replace, a laboratory HbA1c test and is not intended to suggest changes in treatment decisions or to be used as a substitute for professional health care advice.

In the current study, the algorithm was incorporated into the MyStar Extra BG meter and its accuracy examined in a clinical setting. Comparison of the eA1c with the laboratory HbA1c values showed, like the in silico testing, that 97.5% of the eA1c results for subjects with diabetes fell within ±20% of the laboratory HbA1c values. The BG meter is designed to report eA1c in the HbA1c reportable range of 6-10%, with values below 6 and above 10 reported only as “low” and “high,” respectively. If the results are confined to the reportable range, over 99% of the eA1c results were within ±20% of the laboratory HbA1c values. This result is comparable to the accuracy of contemporary SMBG devices,^6^ which means the model-based estimation procedure does not introduce further bias in the estimate, beyond the errors inherent with the inputted SMBG data. This indicates that the HbA1c estimation feature of the MyStar Extra BG meter is effectively accurate for the intended users of the device.

The MyStar Extra BG meter also incorporates an eA1c trend arrow designed to aid subjects in knowing how their current therapy is affecting HbA1c in the period between obtaining laboratory HbA1c values. The hypothesis was tested that at least 97% of trend arrows displayed were steady or in the same direction as the change in reference HbA1c. The acceptance criterion was based on the trend arrow analysis obtained during validation of the trend arrow in silico.^5^ While the acceptance criterion specified by the study protocol was not met, 95.4% of the trend arrows for the reportable HbA1c range did not trend opposite to the reference in subjects with diabetes. The data set used to validate the algorithm was representative of subjects who were insulin naïve on oral hypoglycemic agents and treated with insulin glargine or Neutral Protamine Hagedorn human insulin.^5^ The variability of the trend of the HbA1c in that data set was more pronounced compared with the current study. This suggests that the accuracy validation of the trend arrow presented here is representative of a population with minimal glycemic variability.

The MyStar Extra BG meter with the eA1c tool was found to be useful by the majority of subjects, despite the fact that they were blinded to the laboratory-drawn HbA1c result as well as to the eA1c value and eA1c trend arrows calculated by their BG meter. The blinding may have negatively affected the results of the questionnaire by limiting the assessment to only the concept of displaying an A1c estimation. Since the manual and flashcards were written for unblinded meters, the lack of practical hands-on experience made it more difficult for the subject to understand or define “useful.” Having a questionnaire that more accurately depicts the blinded processes and the experiences the subject had with the meter/manual might improve outcomes.

In conclusion, greater than 99% of eA1c values in the reportable HbA1c range of ≥6 to ≤10% from subjects with diabetes fell within ±20% of the laboratory HbA1c. The accuracy of the eA1c feature of the MyStar Extra BG meter was similar to the performance determined in silico. eA1c is intended to raise patients’ A1C awareness by providing an estimated A1c value and trend in between health care professional visits. It is intended as an adjunctive tool to complement, not replace, a laboratory HbA1c test and is not intended to suggest changes in treatment decisions or to be used as a substitute for professional health care advice. The majority of subjects found the tool helpful and agreed that it may motivate them to manage their diabetes better. Real-time SMBG-based estimation of HbA1c has been shown to improve glycemic control.^7^

## References

[1] Koenig RJ Peterson CM Jones RL Saudek C Lehrman M Cerami A Correlation of glucose regulation and hemoglobin AIc in diabetes mellitus. N Engl J Med. 1976;295:417-420. 934240 10.1056/NEJM197608192950804

[2] Manley S Haemoglobin A1c—a marker for complications of type 2 diabetes: the experience from the UK Prospective Diabetes Study (UKPDS). Clin Chem Lab Med. 2003;41:1182-1190. 14598868 10.1515/CCLM.2003.182

[3] Nathan DM Kuenen J Borg R Zheng H Schoenfeld D Heine RJ Translating the A1C assay into estimated average glucose values. Diabetes Care. 2008;31:1473-1478. 18540046 10.2337/dc08-0545 PMC2742903

[4] Trevino G On A1c and its dependence on PG level. Diab Res Clin Pract. 2008;79:e14. 10.1016/j.diabres.2005.11.005 16413629

[5] Kovatchev B Flacke F Sieber J Breton M Accuracy and robustness of dynamical tracking of average glycemia (A1c) to provide real-time estimation of hemoglobin A1c using routine self-monitored blood glucose data. Diabetes Technol Ther. 2014;16:303-309. 24299302 10.1089/dia.2013.0224 PMC3997127

[6] Boren SA Clarke WL Analytical and clinical performance of blood glucose monitors. J Diabetes Sci Technol. 2010;4:84-97. 20167171 10.1177/193229681000400111 PMC2825628

[7] Kovatchev BP Mendosa P Anderson S Hawley JS Ritterband LM Gonder-Frederick L Effect of automated bio-behavioral feedback on the control of type 1 diabetes. Diabetes Care. 2011;34:302-307. 21216860 10.2337/dc10-1366 PMC3024338

